# Freezing point osmometry of milk to determine the additional water content – an issue in general quality control and German food regulation

**DOI:** 10.1186/1752-153X-2-6

**Published:** 2008-03-10

**Authors:** Britta Büttel, Markus Fuchs, Birger Holz

**Affiliations:** 1 Wissenschaftliche Gerätebau Dr. Ing. H. Knauer, Hegauer Weg 38, D-14163 Berlin, Berlin, Germany

## Abstract

**Background:**

The determination of the osmolality of aqueous samples using a freezing point osmometer is a well-established, routine laboratory method. In addition to their use in clinical and pharmaceutical laboratories, freezing point osmometers are also employed in food testing laboratories. One application is the determination of the osmolality of milk. Although cow's milk is a natural product whose water content is approximately 87%, the osmolality of milk is a significant value when the milk is collected from a larger population of animals. This value is used in milk processing to control the water content, based on the German Food Control Regulations for Milk.

**Results:**

Measurement of the freezing point and osmolality of milk samples was performed with a Knauer Semi-Micro Freezing Point Osmometer. Osmolality was measured for the untreated milk samples and following their dilution (by volume) with 10% and 50% water. The measurements were made after 1, 4 and 7 days to evaluate changes over time. All measurement values for the undiluted milk were spread over a small interval with an average of 271 mOsmol/kg. After mixing the milk samples with 10% water, the average decreased to 242 mOsmol/kg, while mixing with 50% water resulted in an average osmolality of 129 mOsmol/kg. There was no significant change for the osmolality within the 7 days (measurements from days 1, 4 and 7).

**Conclusion:**

The results observed demonstrate clearly that the additional water content of milk can be determined easily using a freezing point osmometer. Milk samples that contain additional water have a significantly decreased osmolality, corresponding to an increased freezing point. The effect on osmolality of ageing the milk samples could not be determined in this study's time-dependent measurements.

## Background

The determination of aqueous samples' osmolality using a freezing point osmometer is a well-established, routine laboratory method. *In vitro *diagnosis in the clinical laboratory is regularly based upon blood serum and urine osmolality. Detection of diseases and therapy control are the main targets [1,2]. The freezing point osmometer is also in use in the food laboratory, where its main applications are in the determination of alcohol content in beverages and quality control of other additives [3]. Because the definition of osmolality is the number of solute molecules that are dissolved in 1 kg of solvent (water in this case), in all these applications the solute is that which is determined by osmometry. When milk is measured, its additional water content is the most important factor for the determination of milk osmolality [4,5].

Cow's milk is a natural product and contains many different components including fat, milk sugar, protein, minerals, and approximately 87% water [6]. All components are dependent upon natural deviations determined by the cow's race, feeding, age and other environmental factors. However, the osmolality of milk is a significant value owing to its being highly preserved even when collected from a larger population of animals and pooled before further processing. Osmolality has therefore been used for quality control of this natural product for quite a while [7].

The quality of the milk to be processed further is controlled by the German Food Regulations for Milk [8]. One part of this regulation concerns the water content as measured by its freezing point. Because this parameter is highly preserved, it is convenient to control the quality in a pooled fraction because the result can be transferred to the whole lot. While fat or carbohydrate content can vary within individuals over time as well as in and between populations, water content is a preserved parameter, changed by external factors. Whenever milk processing takes place, additional water may be mixed into the milk. This can occur because of the presence of water in the milking machine pipes or any other kind of dilution with water at the milk producer's site. The freezing point is not to exceed a value of -0.515°C as mentioned in the food regulations concerning milk. If exceeded, the product is not permitted for use in further food processing and has to be discarded owing to its having probably been diluted with water.

This study used several freezing point osmometers to determine the osmolality in aqueous solutions by a specific decrease of the freezing point compared to that of pure water. A depression of the freezing temperature of a milk sample compared to a reference milk sample indicated that the milk sample was contaminated with a soluble component. While most laboratories use only one instrument, in our laboratory five instruments were precisely calibrated for the purpose of this study. Certified solutions [9] together with a number of dilution experiments were used in order to validate measurement equipment and provide an accurate calibration of every instrument included in the study. Blind samples were used to exclude effects originating from packing material, glass vessels and measurement tubes. When the water content of milk is raised, *i.e*. the number of diluted particles per kilogram sample is reduced, this can be identified by an elevation of the freezing point towards 0°C. *Vice versa*, the number of particles dissolved in 1 kg of sample is directly proportional to the decrease of the freezing temperature. This study covers the measurement of undiluted cow's milk taken from conventional packages and dilutions with pure (0 mOsmol/kg) water by 10% or 50% by volume. Dissociation of sample molecules over time was evaluated by re-measurement exactly 4 and 7 days after the undiluted samples had been opened and measured. The aim of this study was to determine exactly the osmolality of real milk samples.

## Results

**Figure 1:**
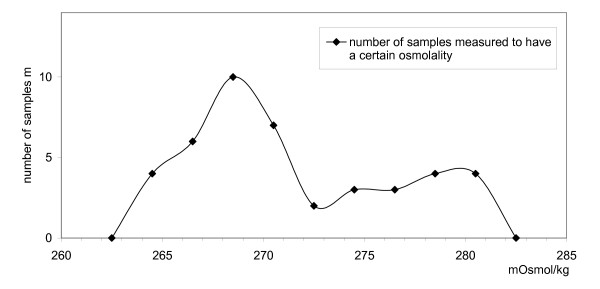
**Milk sample osmolality by sample number**. Number of samples measured to have a certain osmolality.

The distribution of measured osmolalities is shown in Figure 1 (n = 12). The 4 individual measurements give m = 48 measurements of untreated (undiluted) samples.

All individual measurement values were spread within a small interval between 263 and 282 mOsmol/kg. The majority of values were close to 269 mOsmol/kg. The average value of all single measurements was 271 mOsmol/kg. The standard deviation was 5.1 mOsmol/kg, with the whole interval being about 19 mOsmol/kg, which is far less than 10% of the mean.

**Figure 2:**
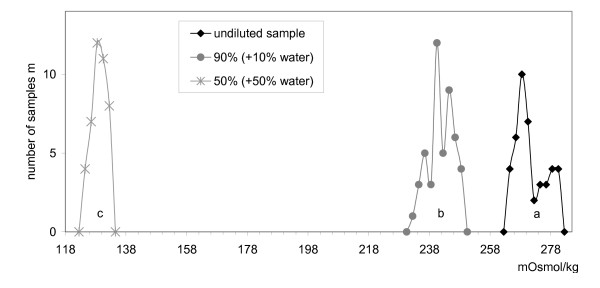
**Dilution of milk samples**. a: undiluted sample; b: 90% (+10% water); c: 50% (+50% water).

Addition of water to the milk sample can be observed when the decrease in the samples' osmolality was measured (Figure 2). Collecting the same data as shown in Figure 1 for the diluted milk samples (b: milk + 10 vol.% water and c: milk + 50 vol.% water) osmolality values showed a significant decrease compared to the undiluted milk (a).

**Figure 3:**
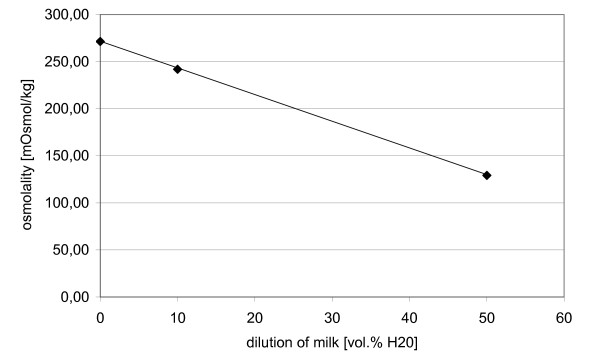
Osmolality of milk as a function of dilution with water.

The average osmolality of undiluted milk samples was 271 mOsmol/kg. After mixing with 10% water by volume, the average decreased to 242 mOsmol/kg. When mixing milk with the same volume of water (50% water) the resulting mean osmolality was 129 mOsmol/kg. The decrease in osmolality effected by dilution was 2.9 mOsmol/kg/1% dilution in the determined range (0% to 50% water) (Figure 3).

**Figure 4:**
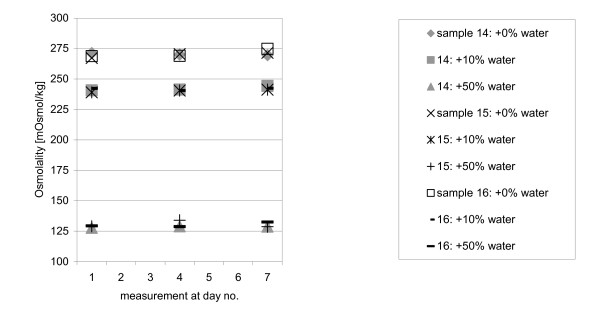
Individual measurements at days 1, 4 and 7.

No significant change in osmolality was observed for all samples 1, 4 and 7 days after opening the packaging, neither for the individual measurement (m = 144) nor for the averaged sample value (n day 1 + 4 + 7 = 36). Figure 4 shows individual measurements for samples 14, 15, and 16. Table 1 shows the average result and standard deviation for each sample. Missing values in Table 1 represent failed measurements, where the milk sample froze too early or did not freeze at all.

**Table 1:** Mean results of all samples

Sample no.	dilution [%] with H_2_O	mean Osmolality [mOsmol/kg]					
		day 1		day 4		day 7	
			standart deviation		standart deviation		standart deviation
1a	0	269,3	1,5	270,8	4,4	268,3	0,6
1b	10	242,3	2,9	241,7	2,5	241,2	2,5
1c	50	131,7	1,5	134,3	0,6	130,8	2,9
2a	0	266,7	2,3	267,0	2,7	272,3	3,3
2b	10	239,0	2,7	237,2	2,8	240,5	1,3
2c	50	130,0	2,9	127,3	1,2	129,0	1,0
3a	0	269,0	0,0	270,0	2,3	272,0	3,0
3b	10	239,7	2,5	240,5	1,9	243,0	2,6
3c	50	132,3	1,2	130,8	3,3	135,3	2,1
7a	0	277,0	1,8	276,3	1,0	275,0	2,0
7b	10	244,0	2,4	244,3	2,8	243,0	0,8
7c	50	129,0	0,8	129,8	3,3	129,5	1,3
8a	0	278,5	1,3	276,0	2,2	278,8	3,4
8b	10	247,3	1,7	243,5	3,1	248,0	2,6
8c	50			130,3	0,6		
9a	0	279,3	2,2	279,5	1,0	277,0	0,8
9b	10	247,8	1,5	245,8	1,3	246,8	1,3
9c	50	129,7	2,3	130,0	2,2	131,5	1,3
11a	0	268,5	3,8	273,6	1,7	278,4	2,1
11b	10	242,3	2,5	243,0	1,4	248,8	0,5
11c	50	129,0	3,9	128,5	2,1	131,3	2,5
12a	0	265,8	1,7	269,3	1,3	274,5	0,6
12b	10	239,7	5,4	237,5	1,0	244,5	1,3
12c	50	127,8	1,7	124,5	0,6	131,5	1,7
13a	0	271,3	0,6	272,5	1,9	275,0	1,4
13b	10	238,6	3,4	241,8	1,5	245,3	1,7
13c	50	127,2	2,6	126,3	1,7	131,8	1,5
14a	0	271,3	2,8	270,0	1,7	269,5	2,1
14b	10	240,7	2,3	241,3	4,0	244,3	4,0
14c	50	127,8	3,6	129,0	3,5	128,8	1,3
15a	0	267,3	2,6	270,3	5,5	271,5	3,7
15b	10	239,0	2,0	240,5	5,4	241,3	3,5
15c	50	129,0	1,8	134,0	1,0	128,7	1,2
16a	0	268,7	2,3	269,0	1,4	274,7	1,5
16b	10	242,3	4,9	240,5	5,1	242,3	2,5
16c	50	129,3	0,6	128,7	3,2	132,5	2,1

## Discussion and Conclusion

From an analytical point of view, milk is a rather complicated sample. Although the content of fat, carbohydrate and other ingredients may vary with the feeding and health status of the animal, the total osmolality value is preserved in a rather small interval. Since the fat content of milk has, if any, only a very small influence on the milk samples' osmolality [10], the results are not discussed with respect to this parameter.

The natural water content of milk only varies to preserve osmolality. This makes it possible to measure additional water content in terms of changes in osmolality. The measurements in this study were made with regular processed unskimmed milk; the same method can be used to determine the additional water content in fresh unskimmed milk or with intermediate products during food processing. Also, partly skimmed milk or milk with reduced fat content can also be measured [11]. All measurement values in this study gave an average of 271 mOsmol/kg. This value is 2.2% less compared to the average recommended by the German Food Regulation (-0.515°C corresponds to 277 mOsmol/kg). This slightly decreased osmolality and increased water content can be explained by the fact that ready packed milk was evaluated in this study.

The results observed demonstrate clearly that the additional water content of milk with different origins can be easily determined using a freezing point osmometer. Milk samples that show additional water content have a significantly decreased osmolality, or an increased freezing point. With the addition of 10 vol.% water, osmolality is increased by 29 mOsmol/kg. This value shows the sensitivity of the method: 2.9 mOsmol/kg per % water, which is rather linear in the range of 0 – 50% additional water (see Figure 3). With higher dilution the activity coefficient reflects the degree of further dissociation and lower salt concentration and makes the effect of additional water content on osmolality more complex, *i.e*. non-linear. However, the method produces stable and significant values for the dilution of milk samples.

The effects on osmolality of ageing milk samples could not be determined in the time-dependent measurements of this study (days 1, 4 and 7, compare Figure 4). According to Chen this is expected for highly concentrated milk samples only [10].

The method described in this paper is a standard method in the German Food Regulation for Milk [8] and the additional water content must be determined according to it. The additional water content and not the total water content are to be determined. This is a relative measurement comparing the results to a calibration solution and can be performed using a simple and easy-to-use Freezing Point Osmometer. If cleaning of the pipelines in the milk processing does not lead to a dilution with cleaning water, but to pollution of the milk with cleaning acid or neutralizing agents (residual salts), this could also be determined by measuring the freezing point of the milk – a depression of the freezing point corresponds to an increase in osmolality.

Alternative measurement methods for water content are mainly used for determining residual amounts of water in milk products along the food processing line, such as cheese, butter or food made of milk or milk products. The total water content of these samples is determined, for instance, by the Karl-Fischer titration, pulsed NMR spectroscopy, or gas humidity measurements using phosphorus pentoxide or drying in the infrared beam. These methods are not suitable for determining the additional water content of milk, because they cannot be applied to samples with high total water content, such as milk.

## Experimental

Measurement of the freezing point and osmolality of milk samples were performed with the KNAUER Semi-Micro Osmometer K-7400 (KNAUER, Berlin, Germany, Order No. A3707) as a stand-alone instrument (instrument #1) or together with EuroOsmo 7400 control and data acquisition software (Order No. A3705; instrument #2). Sample vials were made of glass (Order No. A0913), and calibration solutions were either 300 mOsmol/kg (Order No.Y1240) or 400 mOsmol/kg (Order No.Y1241) solution consisting of sodium chloride in water. Cleaning solution (Order No. Y1277) was used for cleaning the measurement head. Instruments #3, #4, and #5 were used as reference instruments and were calibrated with a urine control solution (Bio-Rad, Redmond, CA, USA, Order No. 435).

Before starting a series of measurements, the Freezing Point Osmometers K-7400 were calibrated using pure water (osmolality 0) and one of the calibration solutions, either 300 mOsmol/kg or 400 mOsmol/kg. The instrument was set to display the freezing point temperature instead of the osmolality result (cryostat mode). The results of the calibration are shown in Table 2.

**Table 2:** Freezing point of water and calibration solution

Calibration Solution [mOsmol/kg]	Freezing Point Temperature [°C]
0	-0.000
300	-0.557
400	-0.743

Exactly 150 μl of the calibration solution or the milk sample were measured into a glass vial using an Eppendorf pipette. The measurement vial was then carefully placed over the thermistor and head wire on the measurement head. After starting the measurement, the run proceeded and terminated automatically. The sample was cooled down slowly below the freezing point and if a previously selected temperature was reached, the wire was moved into the vial to initiate freezing. The crystallisation heat raises the temperature immediately until the freezing temperature has been reached. When the temperature curve shows a plateau, this freezing point temperature was taken as the measurement value and the concentration in mOsmol/kg was calculated based upon the calibration curve stored in the instrument. The final concentration was displayed on the software interface.

Between different samples, the measurement head thermistor was carefully wiped with a lint-free paper towel. After 4 repeat measurements with one sample, the thermistor and the wire of the measurement head were washed with distilled water (maximum allowed osmolality = 2) and then with diluted cleaning solution and again with distilled water. The measurement head was dried with a lint-free paper towel or air-dried before starting the next measurement.

The samples were taken from 12 conventional cow's milk packages, eleven of these in paper-aluminum packages and one in a glass bottle. Three fresh milk diary products (pasteurized milk) and nine long-life milk products (UHT milk) were randomly chosen for this study. The fat content in the fresh milk varied from 3.5% to 3.8% (w/v), while long-life milk had 3.5% for all samples tested. Nine registered milk producers were the manufacturers of the tested products. The osmolality of the milk samples was measured for the untreated milk and when diluted with 10% and 50% pure water by volume.

To obtain a 10% vol. dilution, 4.6 ml of the corresponding milk sample and 0.4 ml of pure water were mixed in a 5 ml glass vessel. To exclude effects from the glass vessel, one additional vessel was filled with 5.0 ml of water, closed and treated in the same way as the samples. This extra water sample was proven to have 0 or 1 mOsmol/kg after the same incubation time. Every sample was shaken for 1 min after capping with a PTFE coated septum/aluminum cap and stored into the refrigerator for 10 min before measurement. A 50% sample was prepared by mixing 2.5 ml of milk with 2.5 ml of water and treated in the same way. To avoid foam, the samples were shaken carefully. The volumes 4.6 and 2.5 ml were pipetted using a glass syringe that had been cleaned with pure water before use.

A measurement value was taken for days 1 (maximum 30 min after opening the package), 4 and 7 to evaluate changes over time. Between measurements, the milk was stored in a refrigerator at 4°C. Every sample at every dilution and time interval was measured about four times.

## Authors' contributions

The authors contributed equally to this work.
